# Preclinical evaluation of CAR-T cell immunotherapy with a fully human EpCAM-specific scFv against pancreatic cancer

**DOI:** 10.1007/s00262-025-04267-x

**Published:** 2025-12-19

**Authors:** Ying-Ying Fan, Ya-Ling Liu, Xiao-Fan Liu, Si-Qi Jiang, Hui Yang, Hao-Ran Zhang, Ping Wei, Qian-Rong Huang

**Affiliations:** 1 National Vaccine & Serum Institute (NVSI), Beijing, 101111 China; 2 National Engineering Research Center for Novel Vaccine, Beijing, 101111 China; 3 Center for Cell and Gene Circuit Design, CAS Key Laboratory of Quantitative Engineering Biology, Shenzhen Institute of Synthetic Biology, Shenzhen Institute of Advanced Technology, Chinese Academy of Sciences, Shenzhen, 518055 China; 4 Center for Quantitative Biology and Peking-Tsinghua Joint Center for Life Sciences, Academy for Advanced Interdisciplinary Studies, Peking University, Beijing, 100871 China

**Keywords:** EpCAM, Chimeric antigen receptor T cells, Pancreatic cancer, Immunotherapy

## Abstract

Pancreatic cancer is an aggressive malignancy with poor prognosis. Recent advances in chimeric antigen receptor (CAR)-T cell immunotherapy have offered renewed hope. The pivotal factors determining the effectiveness of CAR-T cells in the clinic is the selection of a reliable target and a specific ectodomain of CAR molecule. EpCAM is widely expressed in epithelial tumors, including pancreatic cancer, and is used as a tumor target in clinical trials of CAR-T cell therapy for gastric and colorectal cancers. However, the feasibility of using EpCAM CAR-T cells in pancreatic cancer still needs to be verified. In this work, we reported novel EpCAM CAR-T cells with a fully human single-chain variable fragment and evaluated their cytotoxic effects in a panel of pancreatic adenocarcinoma cell lines, three-dimensional tumor spheroid models and patient-derived organoids, and two xenograft mouse models. We demonstrated that EpCAM CAR-T cells exhibited potent and specific anti-tumor activity against EpCAM positive pancreatic cancer in vitro and in vivo, without adverse effects by systematic delivery in xenograft mouse models. Our preclinical results provided evidence of the efficacy and feasibility of EpCAM CAR-T cells for the immunotherapy of pancreatic cancer.

**Supplementary Information:**

The online version contains supplementary material available at 10.1007/s00262-025-04267-x.

## Introduction

Pancreatic cancer, of which approximately 90% is pancreatic ductal adenocarcinoma (PDAC), remains one of the most aggressive and treatment-resistant malignancies, with a dismal 5-year survival rate of less than 10% [1]. PDAC exhibits pathological features, including dense desmoplastic stroma, abundant cancer-associated fibroblasts and extracellular matrix, disorganized vasculature, and a profoundly immunosuppressive microenvironment [2]. These characteristics collectively create physical and biological barriers that severely restrict immune cell infiltration and function. The resulting paucity of tumor-infiltrating effector T cells directly correlates with the characteristically poor response of PDAC patients to immunotherapies, particularly immune checkpoint blockade [3–5]. Adoptive cell therapy using tumor-specific T cells has emerged as a promising strategy to overcome both the infiltration barrier and functional impairment of endogenous immune cells.

Chimeric antigen receptor (CAR)-engineered T cells represent a breakthrough in targeted immunotherapy, the synthetic ectodomain of which enabling precise recognition of tumor-associated antigens (TAAs) [6, 7]. Since the first FDA approval in 2017, seven CAR-T cell therapies have been commercialized as advanced therapy medical products (ATMP), demonstrating remarkable efficacy against hematological malignancies. However, their application in pancreatic cancer yet has shown very limited clinical efficacy. Available clinical trial data (Table 1) reveal that the majority only exhibited short-term responses or disease progression after treatment with CAR-T cells with the investigational targets, including human epidermal growth factor receptor 2 (HER2) [8], CD133 [9], mesothelin [10, 11] and epidermal growth factor receptor (EGFR) [12]. While CAR-T cells with the emerging antigen Claudin18.2 has achieved prolonged tumor regression with a favorable response rate [13–15], which suggests that with an appropriate target, CAR-T cell therapy has the potential to be an effective component of clinical therapy for pancreatic cancer. There is still need for exploring new available targets in pancreatic cancer.

**Table 1:** Completed and published CAR-T cell therapy clinical trials in pancreatic cancer

Trial number	Phase	Target	CAR construct	Number of PC patients (total)	Efficacy	References
NCT01935843	I	HER2	Anti-HER2 scFv-CD137–CD3*ζ*	2 (11)	SD 2	[8]
NCT02541370	I	CD133	Anti-CD133 scFv–CD8*α*–CD137–CD3*ζ*	7 (23)	PR 2 SD 3 PD 2	[9]
NCT01897415	I	MSLN	Anti-MSLN scFv (SS1)-4–1BB–CD3*ζ*	6 (6)	PD 4 SD 2	[10]
NCT02159716	I	MSLN	Anti-MSLN scFv (SS1)–4-1BB–CD3*ζ*	5 (15)	PD 3 SD 2	[11]
NCT01869166	I	EGFR	Anti-EGFR scFv–CD8*α*–CD137–CD3*ζ*	16 (16)	PR 4 SD 8 PD 2 Loss of follow-up 2	[12]
NCT03874897	I	CLDN18.2	Anti-CLDN18.2 scFv–CD8*α*–CD28–CD3*ζ*	10 (98)	PR 2 SD 7 PD 1	[13, 14]

SD, stable disease; PR, partial response; PD, progressive disease

The epithelial cell adhesion molecule (EpCAM) is a transmembrane glycoprotein that is constitutively expressed in epithelial tissues and frequently overexpressed in epithelial-derived malignancies, including lung carcinoma, colorectal cancer, and pancreatic cancers [16, 17]. Notably, EpCAM demonstrates elevated expression in cancer stem cells and circulating tumor cells, where it plays a pivotal role in tumor recurrence and metastatic dissemination [18, 19]. Preclinical studies consistently show EpCAM CAR-T cells inhibit tumor progression in animal models of colorectal cancer [20–24], gastric cancer [25–27], prostate cancer [28] and ovarian cancer [20, 29]. Two early phase clinical trials (NCT02915445, NCT05028933) further confirm therapeutic activity in gastric and intestinal cancers [23, 30]. These findings collectively establish EpCAM as a clinically actionable target for CAR-T cell immunotherapy and there still lacks of comprehensive preclinical investigations of EpCAM CAR-T cells in pancreatic cancer.

**Fig. 1:**
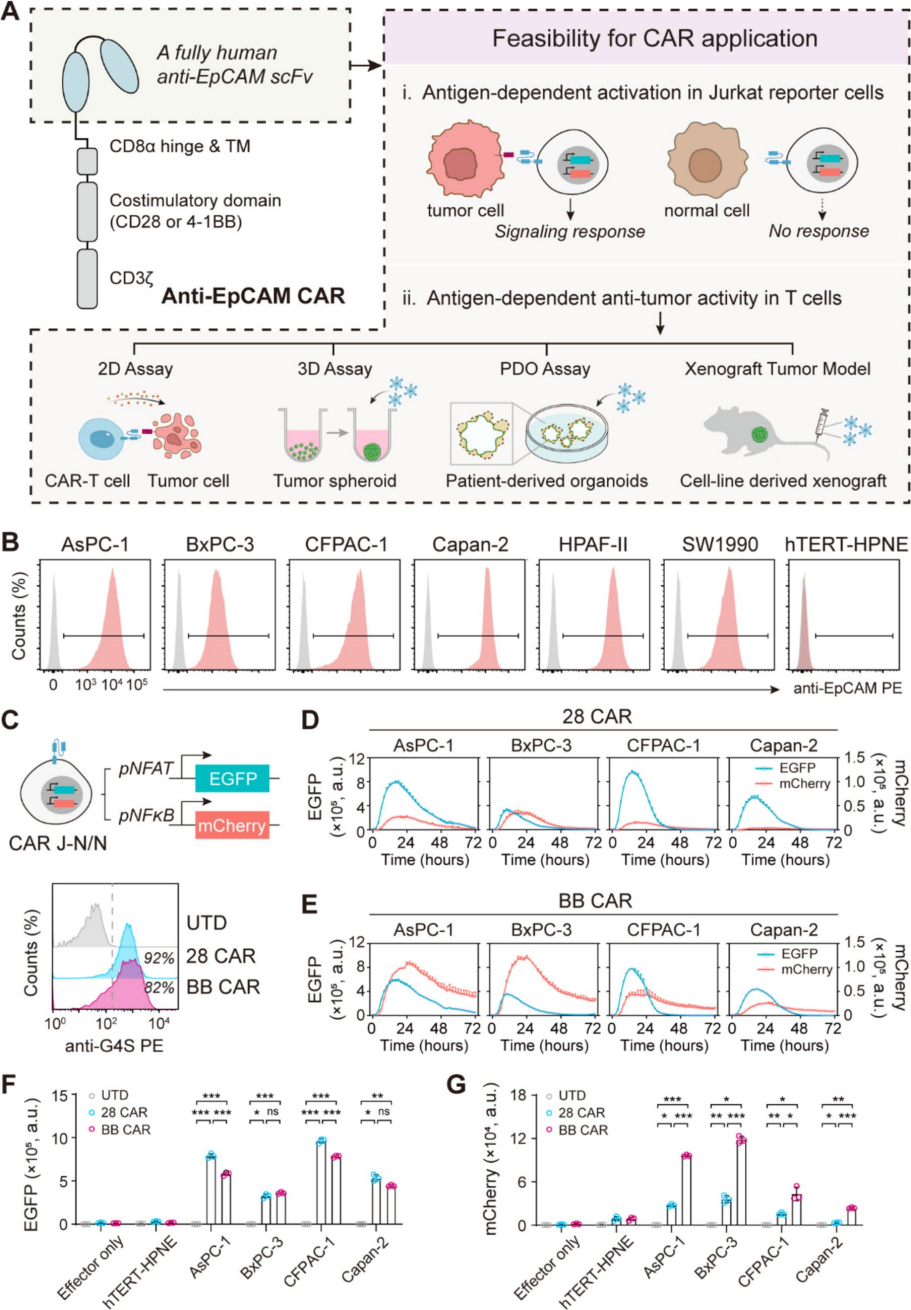
Preclinical investigation of a novel anti-EpCAM CAR for pancreatic cancer **A** Schematic diagram of developing a novel antibody for CAR construction and application against tumor. The anti-EpCAM CAR constructs used in this study contained a fully human anti-EpCAM scFv, CD8α hinge and transmembrane, costimulatory domain of CD28 or 4-1BB and CD3ζ, to generate 28 CAR and BB CAR, respectively. The two CARs were transduced into J–N/N to test antigen-induced signaling response and primary T cells to evaluate antigen-dependent anti-tumor activity using different models, including 2D assay, 3D assay, PDO assay and xenograft tumor models. **B** Representative overlays of the flow cytometry showed that high-level cell surface expression of EpCAM (red) compared to control (grey) in six human pancreatic adenocarcinoma cell lines and no expression in normal human pancreatic ductal cell line, hTERT–HPNE, which was determined by staining with PE labelled anti-human EpCAM antibody (1B7). **C** Schematic representation of dual-color reporter cells named J–N/N, with NFAT inducible EGFP and NF-κB inducible mCherry reporter genes incorporated into Jurkat cell line. Representative flow cytometry histogram plots detailing 28 CAR and BB CAR expression on the surface of J–N/N cells, as determined by staining with an anti-G4S antibody. **D, E** Signaling dynamics of 28 CAR (**D**) and BB CAR (**E**) J–N/N T cells after co-culture with four EpCAM positive pancreatic adenocarcinoma cells at a 5:1 ratio for 72 h. Data at each timepoint are presented as mean with SD of triplicate wells. **F, G** Peak of GFP intensity (**F**) and RFP intensity (**G**) in Fig. 1D, E was extracted. Effector only and EpCAM-negative hTERT–HPNE as negative control. Data are presented as mean with SD of triplicate wells (unpaired *t* test; ns, not significant, *P < 0.01, **P < 0.001, ***P < 0.0001)

Here, we reported novel EpCAM CAR-T cells with a fully human single-chain variable fragment (scFv) and evaluated their specific cytotoxic effects in pancreatic cancer. To verify the feasibility of using this targeting component to construct CAR molecules, we generated two second-generation CAR constructs with commonly used CD28 or 4-1BB. Then, the two CARs were transduced into Jurkat reporter cells to compare antigen-induced signaling response and primary T cells to evaluate antigen-dependent anti-tumor activity by using multiple models, including two-dimensional (2D) assay, three-dimensional (3D) assay, patient-derived organoids (PDOs) assay, and xenograft tumor models (Fig. 1A). First, the EpCAM CAR could elicit antigen-dependent signaling activation in Jurkat reporter cells. Second, the EpCAM CAR-T cells could elicit antigen-dependent proliferation, cytokine secretion and cytotoxicity to target tumor cells in vitro in both two-dimensional and three-dimensional pancreatic tumor models. Furthermore, adoptive transfer of EpCAM CAR-T cells could significantly cause tumor regression in vivo in two xenograft mouse models of pancreatic cancer, without signs of organ damage. These preclinical results provided evidence of the efficacy and feasibility of EpCAM CAR-T cells for the immunotherapy of pancreatic cancer.

## Materials and methods

### Vector construction

The CAR constructs consisting of CD8α signal peptide, EpCAM-specific scFv, CD8α hinge and transmembrane, the cytoplasmic domain of human CD28 (for 28 CAR) or 4-1BB (for BB CAR) and CD3*ζ* were synthesized by a vendor (Tsingke, China). EpCAM-specific scFv was derived from a fully human anti-EpCAM monoclonal antibody called PR001801 and based on the configuration of the variable heavy and light chain, spaced by a (G4S)_3_ linker. The antibody sequence was disclosed and extensively described in granted patent application number CN110950959B. The CAR sequences were subcloned into a lentivirus vector with EF1α promoter using T4 DNA ligase (NEB) after digestion with restriction enzymes XbaI and MluI (NEB), following the manufacturer’s recommended protocol. The fluorescent reporter genes were synthesized by a vendor (Tsingke, China) and subcloned into lentivirus vectors using same strategy with CAR constructs. All sequence of the final constructs was verified by Sanger sequencing (Tsingke, China).

### Cell culture

Human embryonic kidney (HEK) 293 T, human pancreatic adenocarcinoma cell lines including AsPC-1, BxPC-3 and derived GFP expressed cell lines were grown in Dulbecco’s modified Eagle’s medium (DMEM, Gibco) with 10% fetal bovine serum (FBS), 100 U/mL penicillin–streptomycin (Solarbio). Capan-2 and derived GFP expressed cell lines were grown in McCoy’s 5A medium modified (Gibco) with 10% FBS and 100 U/mL penicillin–streptomycin (Solarbio). CFPAC-1 and derived GFP expressed cell lines were grown in Iscove’s modified Dulbecco’s medium (Gibco) with 10% FBS and 100 U/mL penicillin–streptomycin (Solarbio). HPAF-II cells were grown in Eagle’s minimum essential medium (ATCC) with 10% FBS and 100 U/mL penicillin–streptomycin (Solarbio). The normal human pancreatic ductal cell line, hTERT–HPNE and derived GFP expressed cell lines were grown in 70% DMEM without glucose (Gibco) and 25% medium M3 base (Incell) supplemented with 5% FBS, 10 ng/mL human recombinant EGF, 5.5 mM D-glucose and 750 ng/mL puromycin. SW1990 and Jurkat reporter cell line (hereinafter referred to as J–N/N) were grown in RPMI-1640 medium (Gibco) with 10% FBS and 100 U/mL penicillin–streptomycin (Solarbio). Human peripheral blood mononuclear cells (PBMCs) were cultured in X-VIVO 15 medium (Lonza) supplied with 200 IU human IL-2 (Four Rings), hereafter referred to as cX-VIVO. All cells were grown at 37 °C and 5% CO_2_ with saturating humidity. The resource of cell lines used in this study is specified in Table S1.

### Lentivirus production

HEK293T cells were cotransfected with the lentiviral vector plasmids and lentiviral packaging plasmids psPAX2 and pMD2.G by calcium phosphate cell transfection method. Cell culture media containing viral particles were harvested at 48 and 72 h after transfection, spun down at 500 g for 5 min to remove cellular debris, concentrated by adding sterile 25% (w/v) PEG8000 and 750 mM NaCl directly to the supernatant to final concentration of 5% (w/v) PEG8000 and 150 mM NaCl and then incubated at 4℃ overnight. The lentivirus was spun down at 4,000 g for 20 min and resuspended with PBS or cX-VIVO medium. The virus was stored at − 80 °C before usage.

### Generation of GFP or CAR expressed cell lines

All stable cell lines used as target in killing assays were generated by lentiviral infection. AsPC-1-G-L, BxPC-3-G-L, CFPAC-1-G-L, and Capan-2-G-L cells were transduced by a lentivirus that expressed Luciferase and copGFP, then grown in medium with puromycin. hTERT–HPNE–GFP were transduced by a lentivirus that expressed copGFP and purified based on GFP expression using flow cytometer. CAR J–N/N cells were generated by transducing J–N/N cells using the lentiviral constructs of 28 CAR and BB CAR. All lentiviral vectors used in this study shown in Table S1.

### Primary human T cell isolation, activation and transduction

Human pan T cells were isolated directly from PBMC samples by using magnetic negative selection. Briefly, 10 million PBMCs were labeled with 10 μL Pan T Cell Biotin–Antibody Cocktail (Miltenyi) in 40 μL MACS buffer (PBS supplemented with 0.5% BSA and 2 mM EDTA) at 4 °C for 5 min. Then, added 30 μL MACS buffer and 20 μL Pan-T Cell Microbead Cocktail (Miltenyi), mixed and incubated at 4 °C for 10 min. Unlabeled pan T cells were separated by using MACS LC Columns (Miltenyi) and MACS Separators (Miltenyi) according to the manufacturer’s instructions.

Purified CD3 + T cells were cultured in cX-VIVO medium and stimulated with Dynabeads Human T-Activator CD3/CD28 (Thermo Fisher Scientific) at a bead-to-cell ratio of 1:1, at a concentration of 1 million/mL. On the next day, T cells were incubated with the virus, supplemented with 6 μg/mL polybrene, for 18 h before fresh cX-VIVO medium replacement. At day 4 after T cell stimulation, Dynabeads were removed. Medium was refreshed every 2–3 days. T cells were expanded until days 10–14, when they were rested and could be used in assays.

### Antibody and staining for flow cytometry

For cell-surface staining, cells were incubated with antibodies at 4 °C for 30 min in the dark. Samples were acquired on a two-laser (488, 633 nm) FACSCanto II (BD) or a six-laser (375, 405, 488, 561, 638, 808 nm) CytoFLEX LX (Beckman) flow cytometer. All flow cytometry data were analyzed using FlowJo v.10 or CytExpert v.2 software. The details of antibodies are provided in Table S1.

### Dual-reporter activation time course

To monitor the Jurkat dual-reporter cell line J–N/N activation, CAR transduced or untransduced J–N/N cells were co-cultured with different target cells in a 96-well plate at a 5:1 E:T ratio. The plates were then transferred into an Incucyte S3 live-cell imaging system (Sartorius) to perform time-lapse imaging. Images of bright field, GFP and RFP were captured every 2 h for 3 days. Integrated GFP and RFP intensity were analyzed to represent the dual-reporter activation level of J–N/N cells in the well.

### Proliferation assay

CAR-T cells were stained in PBS with 10 μM Cell Trace CFSE (Thermo Fisher) at 37 °C for 20 min. Then, added five times the original staining volume of cX-VIVO medium to the cells and incubated for 5 min. Cells were pelleted by centrifugation and resuspended in cX-VIVO medium. Stained CAR-T cells were co-cultured with different target cells in a 24-well plate at a 1:1 E:T ratio. After 5 days, CFSE dilution was measured by flow cytometry.

### In vitro CAR-T cytotoxicity

For the killing assay, GFP-positive target cells were seeded in a 96-well plate at 5000 cells/well. Next day, CAR-T cells or T cells were co-cultured with different target cells at an E:T ratio of 4:1, 2:1, 1:1, 1:2 or 1:4, including BxPC-3-G-L, AsPC-1-G-L, CFPAC-1-G-L, Capan-2-G-L, and hTERT–HPNE–GFP. In addition, target cells only set as the negative control. The plates were then transferred into the Incucyte S3 live-cell imaging system (Sartorius). Images of bright field and GFP were captured every 4 h for 3 days. Integrated GFP intensity was analyzed to represent the amount of target cells in the well. GFP intensity of each well was normalized by the initial value. The percentage of cytotoxicity at one point (24, 48 or 72 h) was calculated by the following formula: (1-normalized GFP intensity of effector cell group/normalized GFP intensity of target cell group) × 100%.

### Tumor spheroids killing

For the single spheroid cytotoxicity assay, BxPC-3-G-L cells were seeded in a 96-well ultralow attachment plate (Corning) at 3000 cells/well. For the mixed spheroid cytotoxicity assay, EpCAM positive BxPC-3-G-L cells were pre-mixed with EpCAM negative hTERT–HPNE–RFP cells at a ratio of 5:1. The mixed cells were seeded in a 96-well ultralow attachment plate (Corning) at 3000 cells/well. The plates were centrifuged for 10 min at 150 g and kept still at 37 °C for 1–2 days to allow spheroids to form. Effector cells were added into spheroid containing wells with different E:T ratios (4:1, 2:1, and 1:1) as indicated in the figure texts. The plates were then transferred into the Incucyte S3 live-cell imaging system (Sartorius). Images of bright field, GFP and RFP were captured every 4 h for 3 days. Integrated GFP or RFP intensity was analyzed to represent the amount of target cells in the well. GFP or RFP intensity of each well was normalized by the initial value. The percentage of cytotoxicity at 24 or 72 h was calculated by the following formula: (1-normalized GFP intensity of effector cell group/normalized GFP intensity of target cell group) × 100%.

### Cytokine production

To measure cytokine production, CAR-T or T cells were washed and resuspended in fresh X-VIVO medium without IL-2 addition. Then, CAR-T or T cells were co-cultured with different target cells at a 4:1 E:T ratio in a 96-well plate. 24 h later, the supernatant was collected for cytokine production assay. Measurement of the human IL-2, IL-6, IFN-*γ* and TNF-*α* levels were performed using the Ella immunoassay microfluidic platform (ProteinSimple) according to the manufacturer’s instruction.

### Patient-derived organoid (PDO) model and co-culture assay

Pancreatic cancer metastasis sample and T cells were obtained from the pleural effusion of one patient diagnosed with pancreatic cancer and informed consent was obtained from the patient enrolled in this study. This study was approved by the Ethical Committees of the Yixing People’s Hospital (ethical approval number: 2020-060). Patient-derived organoids were established by K2 Oncology Co., Ltd. according to a previous report [31]. T cells from the patient were stimulated and transduced with lentivirus to generate CAR-T cells. Then, CAR-T cells or T cells were co-cultured with PDOs at the E:T ratio of 10:1 in a 96-well plate. Five days later, the supernatant was collected for cytokine production assay. Measurement of the human IFN-*γ* and Granzyme B levels were performed using the ELISA kits (MultiSciences) following the manufacturer’s instruction. The cytotoxicity of CAR-T cells against PDOs were measured using a human lactate dehydrogenase (LDH)-Cytox Assay Kit (Biolegend) according to the manufacturer’s instruction.

### Mouse tumor xenograft models

Animal experiments were approved by the Institutional Animal Care and Use Committee (IACUC) of the National Vaccine and Serum Institute (NVSI) (No. NVSI–RCD–JSDW–ER-2024158, No. NVSI–RCD–JSDW–ER-2024375) and conducted under the regulations for the administration of affairs concerning experimental animals of China (2017). Two mouse tumor xenograft models were used in this study. BxPC-3-G-L cells (1 × 10^6^) were mixed with 20% Matrigengel matrix (ABW) in 100 μL of PBS and injected subcutaneously (s.c.) into the right flank of C-NKG mice (female, 6 weeks, catalog C001316, purchased from Cyagen, China). Capan-2-G-L cells (1 × 10^6^) were mixed with 10% Matrigengel matrix (ABW) in 100 μL of PBS and injected subcutaneously into the right flank of NOD.Cg-Prkdc^scid^Il2rg^tm1Sug^/ShiJic (NOG) mice (female, 6 weeks, catalog 408, purchased from Vital River, China). Three days later, the mice were divided into four groups based on luminescence values: PBS, UTD, 28 CAR, BB CAR (five mice per group). Un-transduced T cell and two CAR-T cells (5 × 10^6^) in 100 μL of PBS were intravenously (i.v.) injected into the mice. Body weight, tumor size and tumor bioluminescence were weekly recorded. Tumor volume was calculated using the following formula: length × width × width/2.

### Analysis of toxic effects in mice

All mice bearing tumor were euthanized when tumor ulceration and/or tumor volume reached 2000 mm^3^. Blood, tumors and key organs (liver, spleen, lung, kidney and intestine) were collected for evaluation of toxic effect. Serum was separated from blood samples for the blood biochemical test measurement. 4% polyformaldehyde-fixed, paraffin-embedded tumors and key organs were sectioned and stained by hematoxylin and eosin (H&E) to analyse histopathological changes.

### Statistical analysis

All statistical analyses were performed using GraphPad Prism. Student’s *t* test and two-way ANOVA with Tukey’s multiple comparisons test was used to assess significance. The statistical details for each experiment are provided in the associated figure legends.

## Results

### EpCAM CAR signaling activation is antigen-dependent and costimulatory domain determined in Jurkat cells

To test the application of a novel scFv targeting EpCAM for CAR design, we developed a novel EpCAM CAR construct based on the second-generation CAR backbone with a fully human scFv derived from an EpCAM mAb, the costimulatory domain using CD28 (for 28 CAR) or 4-1BB (for BB CAR), and the cytoplasmic domain of CD3*ζ*. The expression levels of EpCAM were evaluated in a panel of human pancreatic associated cell lines by flow cytometry after staining with PE labelled anti-human EpCAM antibody. EpCAM was highly expressed in six different cancer cells, while it was not expressed in the normal pancreatic ductal cell line, hTERT–HPNE (Fig. 1B).

Then, we utilized a dual-color Jurkat reporter cell line to assess the signaling response of this new CAR to EpCAM antigen stimulation (Fig. S1A–C). The Jurkat reporter cells were incorporated with two reporter genes: transcription factor NFAT induced GFP expression and NF-κB induced RFP expression, J–N/N for short, which was a good platform to pre-screen CAR activity [32]. We transduced the two CAR molecules into J–N/N cells, separately. The scFv was based on the configuration of VH–(G4S)_3_–VL, so the CAR expression was determined by staining with the anti-G4S antibody (Fig. 1C). Signaling activity of the 28 CAR and BB CAR in J–N/N cells was assessed by coincubation with a panel of pancreatic adenocarcinoma cells. Both 28 CAR (Fig. 1D) and BB CAR (Fig. 1E) showed GFP and RFP expression in response to four EpCAM positive target cells, but with different signaling strength. By targeting to AsPC-1 and CFPAC-1, 28 CAR induced stronger GFP expression in J–N/N reporter than BB CAR and they induced GFP expression at the same level by BxPC-3 and Capan-2 (Fig. 1F), while the RFP expression of 28 CAR was significantly lower than that of BB CAR in all cell lines (Fig. 1G). Both 28 CAR and BB CAR caused no antigen-independent activation and no response to EpCAM negative hTERT–HPNE. These results indicated that EpCAM CAR signaling activation was generally cell surface antigen dependent and costimulatory domain determined in J–N/N cells.

### EpCAM CAR-T cells exert potent cytotoxic activity against EpCAM positive pancreatic adenocarcinoma cells

**Fig. 2:**
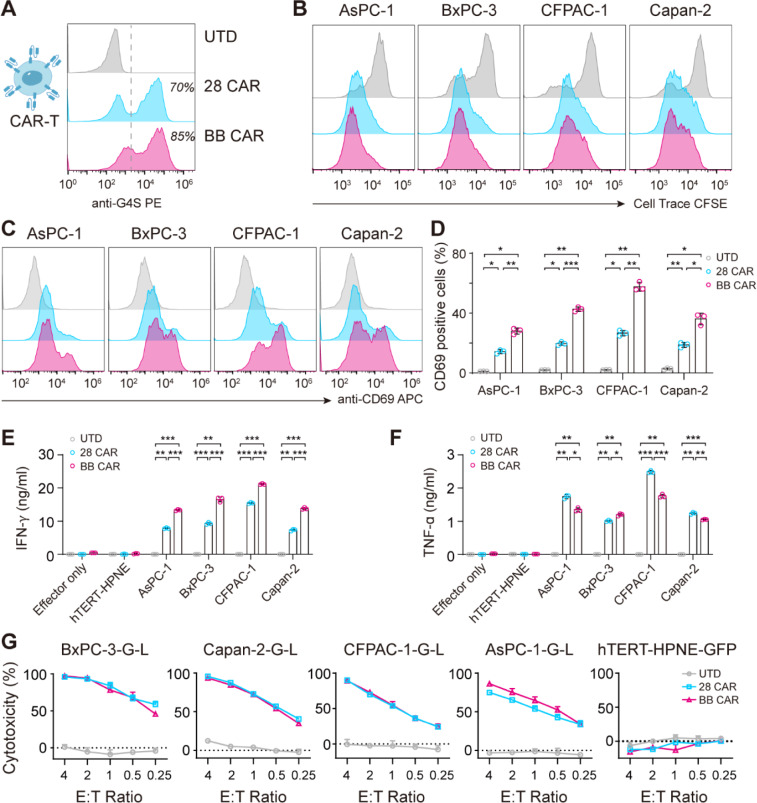
Construction, activation and cytotoxic assays of EpCAM-specific CAR-T cells in vitro. **A** Representative flow cytometry histogram plots detailing 28 CAR and BB CAR expression on the surface of human primary T cells, as determined by staining with an anti-G4S antibody. **B** Flow cytometry results showing the dilution of proliferation staining dye Cell Trace CFSE. Untransduced T (UTD) and two CAR-T cells were stained with 10 μM Cell Trace CFSE and co-cultured with four EpCAM positive pancreatic adenocarcinoma cells at a 1:1 ratio for 5 days before analyzed by flow cytometry. **C** Expression of the activation marker CD69 on UTD and two CAR-T cells after co-culture with target cells at a 4:1 ratio for 24 h by flow cytometry. **D** Quantification of (**C**) showing the percentage of CD69 positive cells. Data are presented as mean with SD of triplicate wells (unpaired *t* test; *P < 0.01, **P < 0.001, ***P < 0.0001). **E, F** IFN-*γ* (**E**) and TNF-*α* (**F**) secretion levels of UTD and two CAR-T cells co-cultured with target cells at a 4:1 ratio for 24 h were measured by Ella kits. Data are presented as mean with SD of triplicate wells (unpaired *t* test; *P < 0.01, **P < 0.001, ***P < 0.0001). **G** Cytotoxic activity of UTD and two anti-EpCAM CAR-T cells against GFP positive BxPC-3, Capan-2, CFPAC-1, AsPC-1 and hTERT–HPNE cells after 48 h of co-culture at E:T ratios of 4:1, 2:1, 1:1, 1:2 and 1:4. Data are presented as mean with SD of triplicate wells. *N* = 1 T-cell donor

Next, we transduced the two CAR molecules into human primary T cells, separately (Fig. 2A). The phenotype analysis showed that both un-transduced T (UTD) and two CAR-T cells were more than 97% CD3 + , with comparable CD4:CD8 ratios, while the CAR-T cells were comprised more effector cells than UTD (Fig. S2A). Then, we detected CAR-T cell activation after exposure to EpCAM positive pancreatic adenocarcinoma cells. A proliferation assay revealed that two CAR-T cells exhibited distinct proliferation after co-cultured with four EpCAM positive pancreatic adenocarcinoma cells (Figs. 2B, S2B). The expression level of the activation marker CD69 was upregulated in two types of CAR-T cells, with BB CAR higher than 28 CAR (Fig. 2C, D). To determine the specific CAR-T cell cytotoxicity and cytokine secretion, four EpCAM positive pancreatic adenocarcinoma cell lines and the EpCAM negative pancreatic cell line were co-cultured with CAR-T or UTD cells. The 24 h co-culture supernatants of E:T (effector to target) ratio 4:1 were collected and detected. Two CAR-T cells showed increased release of IFN*γ*, TNF*α* (Fig. 2E, F), IL-2 and very limited production of IL-6 (Fig. S2C, D) after stimulated by four EpCAM positive pancreatic adenocarcinoma cells while not by hTERT–HPNE. Consistently, two CAR-T cells exhibited potent specific cytotoxicity against four EpCAM positive pancreatic adenocarcinoma cells, while no significant cytotoxic response was observed in hTERT–HPNE (Fig. 2G). The killing efficiency was increased with time and E:T ratio (Fig. S3). Collectively, these data illustrated that EpCAM CAR-T cells were capable of inducing EpCAM specific and potent immune response to pancreatic adenocarcinoma cells.

### EpCAM CAR-T cells show specific cytotoxicity in pancreatic tumor spheroids

**Fig. 3:**
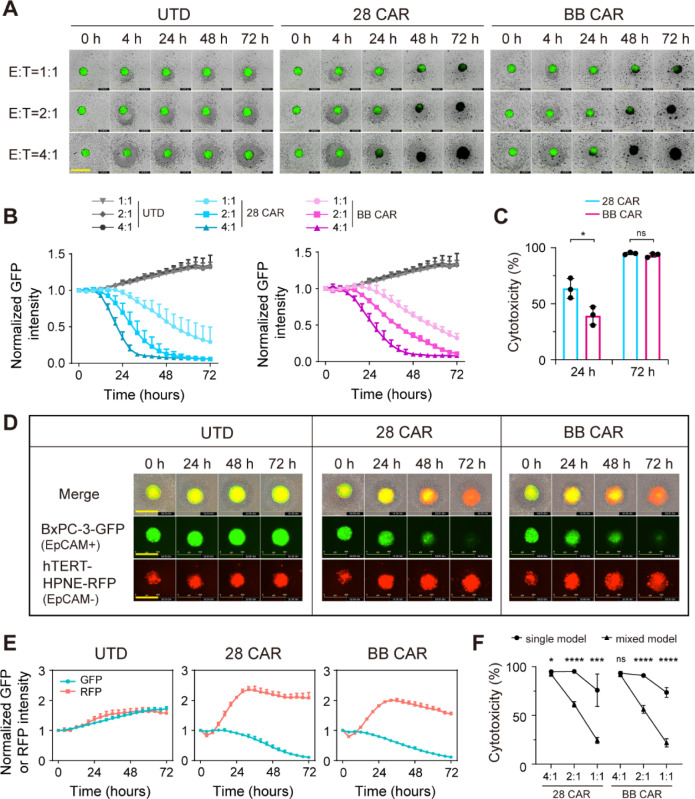
Anti-EpCAM CAR-T cells show specific cytotoxicity in three-dimensional tumoroid model. **A** Representative images from a single tumoroid cytotoxicity assay of GFP positive BxPC-3 cells co-culture with UTD and two anti-EpCAM CAR-T cells at E:T ratios of 4:1, 2:1, and 1:1 for 72 h. Yellow bars indicate 800 μm. **B** Quantification of the resulting tumor reduction dynamics by 28 CAR and BB CAR, as determined by total GFP intensity normalized to the time zero. Data are presented as mean with SD of triplicate wells. **C** Cytotoxic activity of two anti-EpCAM CAR-T cells against single tumoroid of BxPC-3 after 24 and 72 h of co-culture at the E:T ratio of 4:1. Data are presented as mean with SD of triplicate wells (unpaired *t* test; ns, not significant, *P < 0.05). **D** Representative images from a mixed tumoroid cytotoxicity assay of EpCAM positive BxPC-3 cells in green mixed with EpCAM negative hTERT–HPNE cells in red at a ratio of 5:1, co-culture with UTD and two anti-EpCAM CAR-T cells at a E:T ratio of 4:1 for 72 h. Yellow bars indicate 600 μm. **E** Summary of relative GFP and RFP intensity of mixed tumoroid co-culture with UTD and two anti-EpCAM CAR-T cells. Data are presented as mean with SD of triplicate wells. **F** Cytotoxic activity of two anti-EpCAM CAR-T cells against single tumoroid and mixed tumoroid after 72 h of co-culture at E:T ratios of 4:1, 2:1, and 1:1. Data are presented as mean with SD of triplicate wells (unpaired *t* test was performed between single model and mixed model; ns, not significant, *P < 0.05, ***P < 0.005, ****P < 0.001). *N* = 1 T-cell donor

We further established the pancreatic tumor spheroids model and investigated the killing efficiency of EpCAM CAR-T cells in three-dimensional culture systems. In a single tumoroid cytotoxicity assay, which consisted of GFP positive BxPC-3 pancreatic adenocarcinoma cells pre-seeded in the ultra-low attachment plate and formed a single tumor spheroid about 300 ~ 400 μm in diameter, both 28 CAR and BB CAR could efficiently eliminate tumor spheroids compared with UTD (Fig. 3A). The killing dynamics showed that 28 CAR had a faster onset than BB CAR at the E:T ratio of 4:1 with the killing efficiency of 28 CAR at 24 h higher than BB CAR, while their cytotoxicity at 72 h was comparable (Fig. 3B, C and supplementary videos 1–6).

To explore the specificity of EpCAM CAR-T cells, we developed a mixed tumoroid cytotoxicity assay, which was incorporated a uniformly mixture of EpCAM positive BxPC-3 cells in green and EpCAM negative hTERT–HPNE cells in red at a ratio of 5:1. The EpCAM CAR-T cells could efficiently and specifically eliminate EpCAM positive BxPC-3 cells in the mixed tumoroid without killing the EpCAM negative hTERT–HPNE cells (Fig. 3D and supplementary videos 7–9). While the EpCAM CAR-T cells could eradicate BxPC-3-GFP cells in the single tumoroid faster than in the mixed tumoroid model (~ 48 h vs. ~ 72 h) at the E:T ratio of 4:1 (Fig. 3B, E and supplementary videos 10–12). Comparing with the UTD group, the hTERT–HPNE RFP intensity in mixed tumoroid had an evident rise with EpCAM CAR-T cells killing the BxPC-3-GFP cells, probably owing to that target killing loosened the spheroid and made the remaining cell more dispersed. For both 28 CAR and BB CAR, low-dose CAR-T activity was significantly diminished against complex mixed tumoroids relative to the single tumoroids (Fig. 3F), highlighting that physiologically relevant models are critical for quantifying CAR-T function.

### EpCAM CAR-T cells exhibit antitumor functions against human pancreatic cancer organoids

**Fig. 4:**
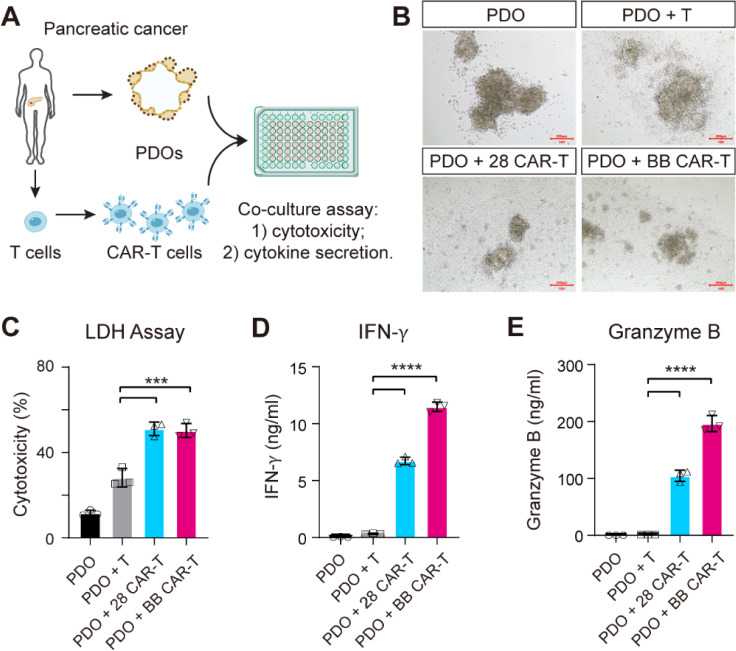
Modelling immunotherapy with co-culture of pancreatic cancer PDOs and CAR-T cells. **A** Schematics of the co-culture experiment workflow. **B** Images of pancreatic cancer PDOs and the co-culture of PDOs with either T cells or CAR-T cells at 5-day endpoint. Scale bar, 200 μm. **C–E** Quantification of LDH release and cytokine production (IFN-γ and Granzyme B) from PDOs after co-culture with effector cells. Data are presented as mean with SD of triplicate wells (unpaired *t* test; *P < 0.05, ***P < 0.001, ****P < 0.0001). *N* = 1 T-cell donor

To evaluate the effects of EpCAM CAR in models closer to the naïve tumor microenvironment, we established a co-culture assay containing PDOs and engineered CAR-T cells using pleural effusion tumor metastasis sample from one patient with pancreatic cancer (Fig. 4A). The PDO models were treated with either T cells or CAR-T cells for 5 days. Compared with T cells, the EpCAM-targeting CAR-T cells accumulated towards the PDOs which underwent deformation and cell lysis (Fig. 4B). The cytotoxic activity was quantitatively analysed by a lactate dehydrogenase (LDH) release kit to further confirm the cell killing of EpCAM CAR-T cells. LDH release was significantly higher in both 28 CAR-T and BB CAR-T cell group than the T cell group (Fig. 4C). Furthermore, the production of IFN-*γ* and granzyme B was significantly increased when EpCAM-targeting CAR-T cells were incubated with PDOs (Fig. 4D, E). These results suggest specific antigen recognition and immune activation during CAR-T cells and PDOs co-culture. Altogether, our data indicate that EpCAM CAR-T cells exhibit antitumor functions against pancreatic cancer PDOs, and further exploring the clinical benefits would be valuable to the pancreatic cancer therapy.

### EpCAM CAR-T cells control tumor growth and exhibit safety in xenograft mouse models of pancreatic cancer

**Fig. 5:**
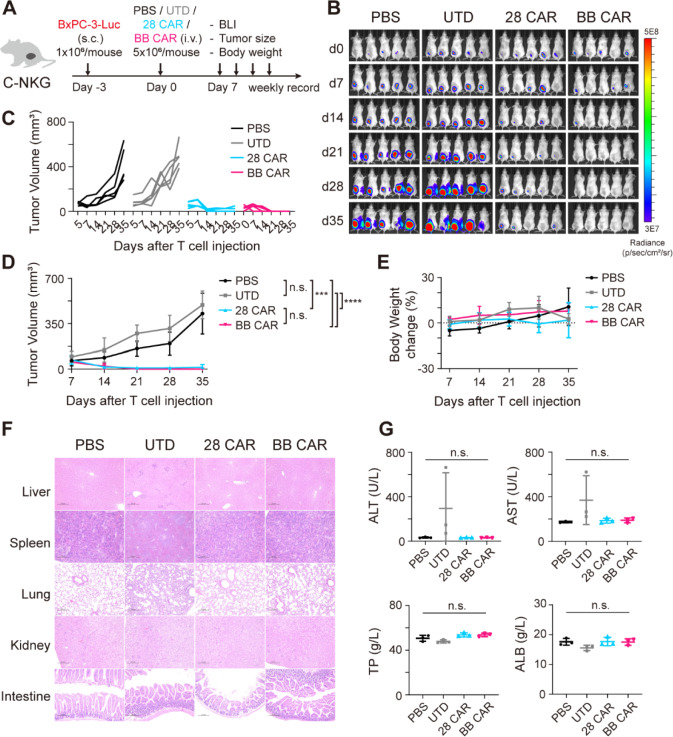
Antitumor efficacy of EpCAM-specific CAR-T cells in xenograft mouse models of BxPC-3 pancreatic cancer. **A** Schematics of the in vivo experiment workflow. C-NKG mice bearing BxPC-3 pancreatic cancer (1 × 10^6^) were intravenously injected with one dose of PBS, UTD, 28 CAR or BB CAR-T cells (5 × 10^6^). After 6–7 weeks of T cell injection, mice were sacrificed to evaluate potential toxic effects of CAR-T cells. **B** Bioluminescence images of tumor burden. **C** Tumor growth curves for individual mice. **D** Average tumor volume measurements over time. Data are presented as mean with SD. *N* = 5 mice per group. (Two-way ANOVA with Tukey’s multiple comparisons test; n.s., not significant, ***P < 0.001, ****P < 0.0001). **E** Average body weight changes after T cell injection. Data are presented as mean with SD. *N* = 5 mice per group. **F** Representative hematoxylin and eosin (H&E) staining images of visceral tissues, including the liver, spleen, lung, kidney and intestine. Scale bars, 200 μm. **G** Organ damage-related serum biomarkers in the liver were measured. Data are presented as mean with SD (unpaired *t* test; n.s., not significant). *N* = 3 mice per group. AST, aspartate aminotransferase; ALT, alanine aminotransferase; TP, total protein; ALB, albumin

To further evaluate the anti-tumor activity and potential toxic effect of EpCAM CAR-T cells, we conducted the in vivo study to validate the findings in vitro. C-NKG mice were inoculated subcutaneously with 1 × 10^6^ BxPC-3-GFP-Luc cells in the right back. Three days later, progressively growing xenografts were confirmed by bioluminescence signal. Mice with established tumors were divided into four groups randomly and received single dose of PBS, UTD, 28 CAR or BB CAR-T cells (5 × 10^6^) intravenously (Fig. 5A). Tumors treated with PBS and UTD cells showed rapid outgrowth. In contrast, 28 CAR and BB CAR-T cells had superior anti-tumor activity, with tumors inhibited in all treated mice, including complete responses (Fig, 5B, C, D).

Moreover, the safety of EpCAM CAR-T cells was preliminarily evaluated. After CAR-T cell treatment, there was no obvious change in body weight in the different treatment groups (Fig. 5E). By 6 weeks, the first death occurred in UTD group and no death occurred in the other three groups. All mice were sacrificed in a week. The livers, spleens, lungs, kidneys and intestines were removed and stained with hematoxylin and eosin (H&E). Blood was collected to detect serum biochemistry values. The staining of tissues showed no pathological signs of tissue damage in the CAR-T cell groups, while the infused control T cells accumulated in the liver and lung, with slight inflammation (Figs. 5F, S4A). The blood biochemical data showed that there was no significant difference between all four groups, but control T cell group had increased aspartate aminotransferase (AST) and alanine aminotransferase (ALT) levels (Fig. 5G), related to liver damage. These differences between CAR-T and control T cells implied that EpCAM CAR-T cells could target and infiltrate the tumor lesions, but the lack of chimeric antigen receptor in control T cells caused deleterious side effects through systematic circulation after intravenous injection.

**Fig. 6:**
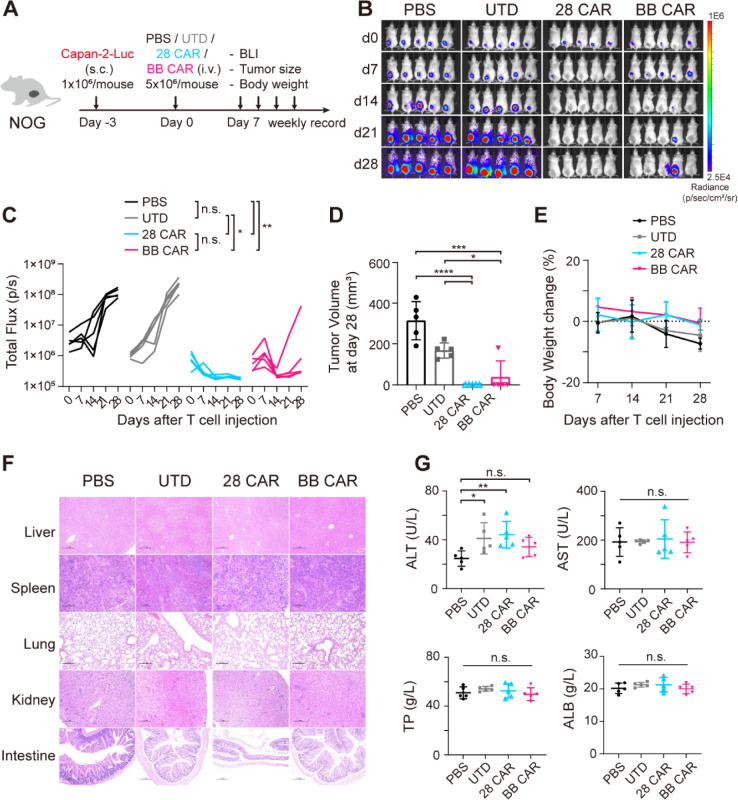
Antitumor efficacy of EpCAM-specific CAR-T cells in xenograft mouse models of Capan-2 pancreatic cancer. **A** Schematics of the in vivo experiment workflow. NOG mice bearing Capan-2 pancreatic cancer (1 × 10^6^) were intravenously injected with one dose of PBS, UTD, 28 CAR or BB CAR-T cells (5 × 10^6^). After 4–5 weeks of T cell injection, mice were sacrificed to evaluate potential toxic effects of CAR-T cells. **B, C** Bioluminescence images and statistical results of tumor burden. (Two-way ANOVA with Tukey’s multiple comparisons test; n.s., not significant, *P < 0.05, **P < 0.01). *N* = 5 mice per group. **D** Tumor volume at 28 days after T cell injection. Data are presented as mean with SD (unpaired *t* test; *P < 0.05, ***P < 0.005, ****P < 0.001). *N* = 5 mice per group. **E** Average body weight changes after T cell injection. Data are presented as mean with SD. *N* = 5 mice per group. **F** Representative hematoxylin and eosin (H&E) staining images of visceral tissues, including the liver, spleen, lung, kidney and intestine. Scale bars, 200 μm. **G** Organ damage-related serum biomarkers in the liver were measured. Data are presented as mean with SD (unpaired *t* test; n.s., not significant). *N* = 5 mice per group

Then, we tested the anti-tumor efficacy and safety of EpCAM CAR-T cells in another highly invasive xenograft model of pancreatic cancer. NOG mice were engrafted subcutaneously with 1 × 10^6^ Capan-2-GFP-Luc cells in the right back. Similarly, 3 days later, progressively growing xenografts were confirmed by bioluminescence signal. Mice with established tumors were divided into four groups randomly and received single dose of PBS, UTD, 28 CAR or BB CAR-T cells (5 × 10^6^) intravenously (Fig. 6A). Again, EpCAM CAR-T cells significantly promoted tumor eradication except one mouse in BB CAR-T cell group (Fig. 6B, C, D). By day 28, two CAR-T cell groups had no change of body mass after treatment but there was a significant body weight loss of mice in PBS group and UTD group compared with those in 28 CAR-T cell group (Fig. 6E). All mice were sacrificed to evaluate safety. The staining of tissues showed no pathological signs of tissue damage in the CAR-T cell groups, while there were lung metastases in the PBS group and the infused control T cells accumulated in the liver and lung, with slight inflammation (Figs. 6F, S4B). There was no organ damage evidence according to blood biochemistry analysis in the EpCAM CAR-T cell groups (Fig. 6G).

Overall, EpCAM CAR-T cells (both 28 CAR and BB CAR) effectively inhibited tumor growth and completely eliminated tumors in two xenograft models of pancreatic cancer BxPC-3 and Capan-2. Meanwhile, no weight loss and no obvious organ damage was observed in the mice treated with EpCAM-specific CAR-T cells. These preclinical investigations demonstrated the anti-tumor efficacy and feasibility of two second-generation EpCAM CAR-T cells against pancreatic cancer.

## Discussion

CAR-T cell therapy utilizes engineered receptors that combine extracellular antigen-targeting domains with intracellular T-cell signaling modules, enabling precise elimination of antigen-expressing cells. Although highly effective in hematologic malignancies and promising for autoimmune diseases [33, 34], solid tumors present challenges due to their immunosuppressive microenvironment and limited tumor-specific antigens, leading to modest clinical outcomes. Nevertheless, recent reports have shown efficacy comparable to CD19 CAR-T in lymphoma, suggesting potential for further development [35]. In a phase I trial [14], 59 patients with heavily pretreated gastric/gastroesophageal junction (GC/GEJ) cancers received a second-generation claudin18.2-specific CAR-T cell infusion and had an overall response rate and a disease control rate of 54.9 and 96.1%, respectively. Besides, the overall survive rate at 12 months was 37.3%. These findings underscore the value of sustained research efforts in this field, with the potential to yield transformative breakthroughs.

The targets of CAR-T cell clinical trials in the treatment of pancreatic cancer patients include EGFR, HER2, CD133, and mesothelin (Table 1). While preliminary results from these studies have demonstrated limited clinical efficacy. The aforementioned Claudin18.2 CAR-T treated 10 patients with pancreatic cancer, an overall response rate and a disease control rate of 20 and 90% were achieved, respectively, with the median duration of responses arriving at 9.4 months [14]. Several additional trials remain ongoing involved other targets, including CEA, MUC1, GD2, ROR1, CD22, CD70, PSCA and some combination targets [36]. The therapeutic effect of these CAR-T cells in pancreatic cancer remains to be verified. EpCAM is a promising pan-cancer target. Some EpCAM-targeting therapies such as oportuzumab monatox and catumaxomab (anti-EpCAM × anti-CD3) show clinical efficacy in EpCAM positive tumors [37, 38]. Preclinically, EpCAM CAR-T cells inhibit growth in colorectal, gastric, prostate and ovarian cancer models. Early trials demonstrate their safety and activity in gastric/colorectal cancers. While EpCAM CAR-T development for pancreatic cancer remains preliminary.

In this study, we comprehensively evaluate the preclinical efficacy in vitro and in vivo of EpCAM CAR-T cells in pancreatic cancer. The novel CAR construct incorporates a human scFv with high specificity for EpCAM. Initial functional validation was performed using a Jurkat dual-reporter cell line J–N/N, where we quantified CAR surface expression levels and antigen-specific signaling activation in response to EpCAM positive pancreatic adenocarcinoma cell lines. The J–N/N system offers several advantages over conventional CAR-T construction, including easier transduction, faster expansion, and shorter validation cycles. Our data demonstrate successful EpCAM CAR expression without baseline signaling activation, eliminating concerns about tonic signaling [39]. Both CAR variants showed antigen-dependent activation when exposed to target cells, with response intensity varying by antigen context. Notably, we observed distinct signaling pathway activation between costimulatory domains—BB CAR induced stronger NF-κB activation than 28 CAR. These findings establish that EpCAM CAR responses are governed by both target antigen characteristics and costimulatory molecule selection.

Building upon these findings, we further generated two EpCAM CAR-T cells and verified the tumor-specific immune response, including cell proliferation, upregulation of surface activation markers, cytokine secretion and cytotoxic activity against pancreatic adenocarcinoma cells. Both 28 CAR and BB CAR demonstrated specific anti-tumor response against EpCAM positive tumor cells while remaining unresponsive to EpCAM negative controls. Despite the differences between 28 CAR and BB CAR in the levels of activation and cytokine secretion, there was little difference in the killing efficiency in vitro. Indeed, the choice of costimulatory molecules have differential effects on T cell function [40]. Some studies suggest that CAR-T cells containing CD28 costimulatory domains exhibit superior antitumor performance, whereas other findings indicate no significant difference or even better results with 4-1BB-based CAR-T cells [41, 42]. The underlying mechanisms have not been fully elucidated and which costimulatory molecule is better for CAR-T function remains inconclusive.

Recent 3D tumor models have opened a new avenue in cancer models, including tumor spheroids and PDOs, which could be used for the testing immunotherapies to assess the enhancement of immune cell infiltration and anti-tumor efficacy against the spheroid targets [43]. Here, we established two 3D tumoroid models, using cell lines aggregates growing in suspension without an extracellular matrix. They have the ability to reproduce the architecture and metabolism of their tissue of origin to a certain extent, while the 2D culture failed to do [44]. In a previous study, Zhang et al. tested their mesothelin targeting CAR-T cells and found this treatment enhanced the anti-tumor response in gastric and ovarian tumoroid models [45]. In this study, we found the EpCAM CAR-T cells could effectively lyse EpCAM-positive cells while causing minimal damage to EpCAM-negative cells in the uniformly mixed tumoroid, indicating their potent and specific cytotoxicity. Nevertheless, the cell line model cannot accurately simulate the complex tumor microenvironment of solid tumors. Hence, we developed a co-culture assay to assess the anti-tumor activity of EpCAM CAR-T cells against the PDOs, which have been found to recapitulate the host tumor genetically and phenotypically. Pancreatic cancer metastasis sample and T cells were obtained from the pleural effusion of one patient diagnosed with pancreatic cancer. It offered a personalized preclinical CAR-T cell testing based on PDO model of pancreatic cancer.

Given the potent anti-tumor activity of EpCAM CAR-T cells in vitro, we explored their efficacy and evaluated the safety in vivo. Two subcutaneous injection xenograft models of pancreatic cancer BxPC-3 and Capan-2 were used. Tumor regression quickly appeared 2 weeks after delivery of EpCAM CAR-T cells intravenously. The efficacy of EpCAM CAR-T cells by systematic delivery pathway suggested the capacity of CAR-T cells to circulate, target and kill tumors. Since there were some reported EpCAM CARs in other tumor models [20–30], we constructed a reported EpCAM CAR composed of a fully human scFv derived from a reported anti-EpCAM monoclonal antibody named UBS54 with a Y6F mutation [27, 46] and evaluated its anti-tumor efficacy together with our EpCAM CAR. The UBS54-Y6F CAR-T cells also exhibited outstanding anti-tumor activity in vitro, both in the 2D assay and 3D assay of pancreatic adenocarcinoma cell lines. However, it failed to inhibit tumor growth in the xenograft tumor model (data not shown). It is not yet understood why there was such a significant difference in the anti-tumor efficacy of these two scFv in vivo.

Align with our findings of EpCAM CAR in vivo, it has been reported that trophoblast cell surface antigen 2 (Trop2) targeting CAR-T cells injected intravenously could inhibit pancreatic tumor growth in the same BxPC-3 xenograft model [47]. Trop2 is an EpCAM family member overexpressed in multiple cancers, including pancreatic cancer. These epithelial markers are promising targets in immunotherapy but their safety has been of concern. A previous study demonstrated that systemically infused murine EpCAM CAR-T cells accumulated in the critical organs and resulted in on-target, off-tumor toxicity [48]. In this preclinical research, we found that no weight loss and no obvious damage was observed in the critical organs in the mice treated with human EpCAM CAR-T cells, indicating that this EpCAM-specific CAR-T cells did not induce deleterious side effects in vivo. However, the immunodeficient xenograft mouse model poorly predicts treatment-related clinical toxicities. There are still enormous differences in target antigen expression profiles, disease and immune status between patients and animal model. For further verification, the EpCAM-humanized immunocompetent mouse model would be necessary for the safety evaluation before translation into clinic. To validate these preclinical results, clinical safety and efficacy assessments should be collected during further Investigator Initiated clinical Trial.

In conclusion, we designed a new EpCAM CAR with a fully human scFv and evaluated the anti-tumor potential in pancreatic cancer. Our preclinical investigations confirm that the EpCAM CAR-T cells showed specific and potent anti-tumor immune responses against pancreatic cancer in vitro and in vivo. Furthermore, adoptively transferred EpCAM CAR-T cells were tolerable in two xenograft models of pancreatic cancer. Therefore, we believe this EpCAM CAR is a promising immunotherapy to the clinic for pancreatic cancer.

## Supplementary Information

Below is the link to the electronic supplementary material.

Supplementary file1 (MP4 962 KB)

Supplementary file2 (MP4 1093 KB)

Supplementary file3 (MP4 1060 KB)

Supplementary file4 (MP4 955 KB)

Supplementary file5 (DOCX 3514 KB)

Supplementary file6 (MP4 1338 KB)

Supplementary file7 (MP4 1309 KB)

Supplementary file8 (MP4 1395 KB)

Supplementary file9 (MP4 1077 KB)

Supplementary file10 (MP4 1174 KB)

Supplementary file11 (MP4 1068 KB)

Supplementary file12 (MP4 1092 KB)

Supplementary file13 (MP4 1082 KB)

## Data Availability

The data that support this study are available from the corresponding author upon reasonable request.
