# Supplementary material for: Preclinical evaluation of CAR-T cell immunotherapy with a fully human EpCAM-specific scFv against pancreatic cancer
Source: Cancer Immunol Immunother. 2025 Dec 19;75(1):22. doi: 10.1007/s00262-025-04267-x (PMC12717346; doi:10.1007/s00262-025-04267-x)
Supplement: Supplementary file 5 — Supplementary file5 (DOCX 3514 KB) [file 262_2025_4267_MOESM5_ESM.docx]

**Supplementary Materials for**

**Preclinical evaluation of CAR T cell immunotherapy with a fully human EpCAM-specific scFv against pancreatic cancer**

Ying-Ying Fan^1,2^, Ya-Ling Liu^1,2^, Xiao-Fan Liu^1,2^, Si-Qi Jiang^3^, Hui Yang^1,2^, Hao-Ran Zhang^1,2^, Ping Wei^3,4,^*, Qian-Rong Huang^1,2,^*

**This PDF file includes：**

Figure S1. to S4

Table S1

Supplementary videos 1-12


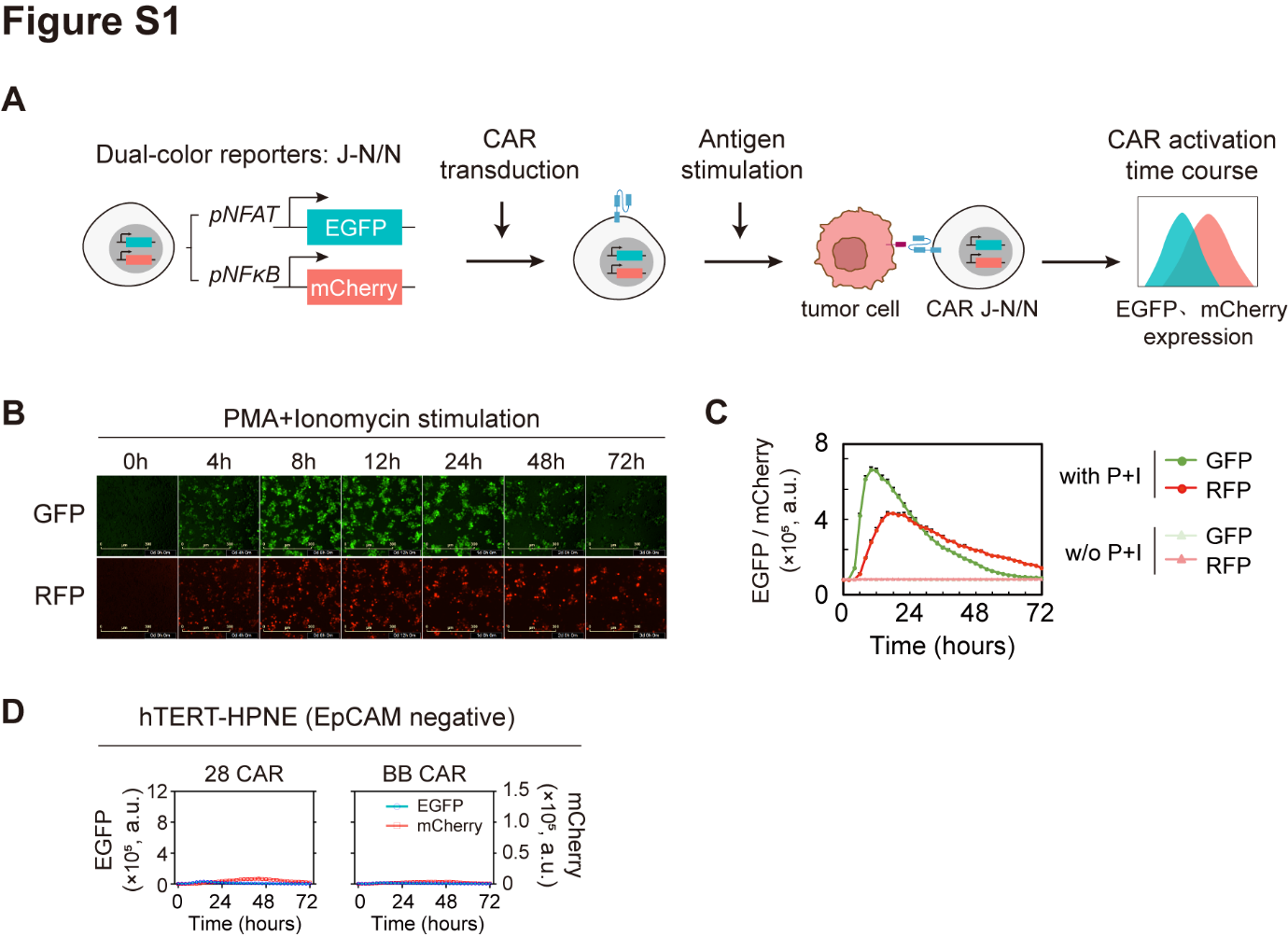


**Figure S1. Anti-EpCAM CAR signaling activation measurement by dual-reporter Jurkat cells.**

(A) Flowchart illustrating the experimental process of evaluating anti-EpCAM CAR signaling activation by the J-N/N cells. (B-C) Integrated GFP and RFP fluorescence intensity of J-N/N cells was recorded every two hours by the Incucyte S3 live-cell imaging system after stimulation with PMA (50 ng/mL) and ionomycin (1 μg/mL). Data at each time point are presented as mean with SE of triplicate wells. (D) Signaling dynamics of 28 CAR and BB CAR J-N/N cells after co-culture with EpCAM negative hTERT-HPNE cells at a 5:1 ratio for 72 hours. Data at each time point are presented as mean with SD of triplicate wells.


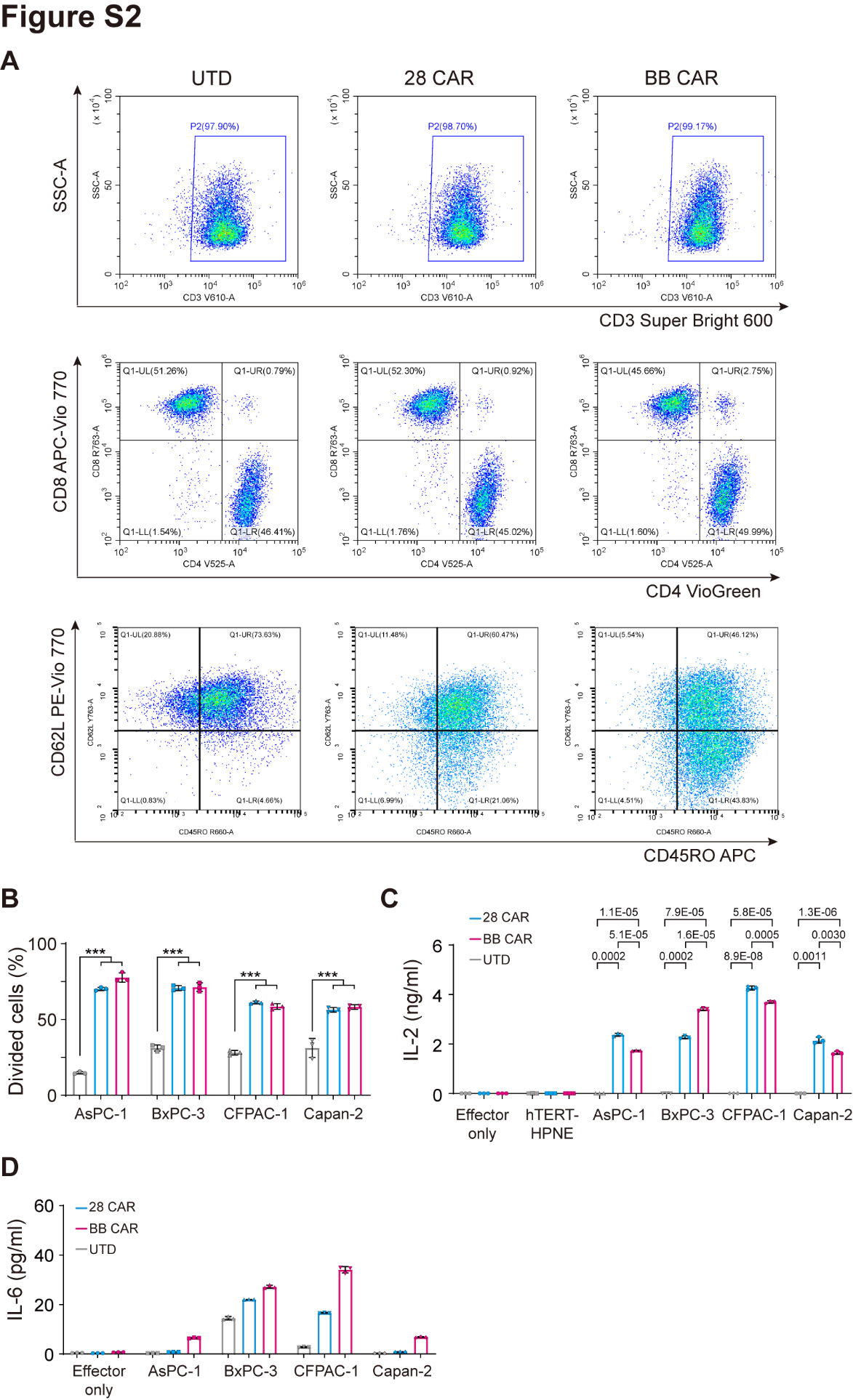


**Figure S2. Immune characteristics and antigenic responses of anti-EpCAM CAR-T cells.**

(A) Representative expression of CD3, CD4, CD8, CD62L and CD45RO markers on UTD and CAR-T cells at 10 days after the transduction of CAR lentivirus. (B) Quantification of Fig.3B showing the percentage of proliferated T cells. Data are presented as mean with SD of triplicate wells (unpaired *t* test; ****P* < 0.005). (C-D) The IL-2 (C) and IL-6 (D) secretion levels of UTD and two CAR-T cells cocultured with target cells at a 4:1 ratio for 24 h were measured by Ella kits. Data are presented as mean with SD of triplicate wells (unpaired *t* test). *N* =1 T-cell donor.


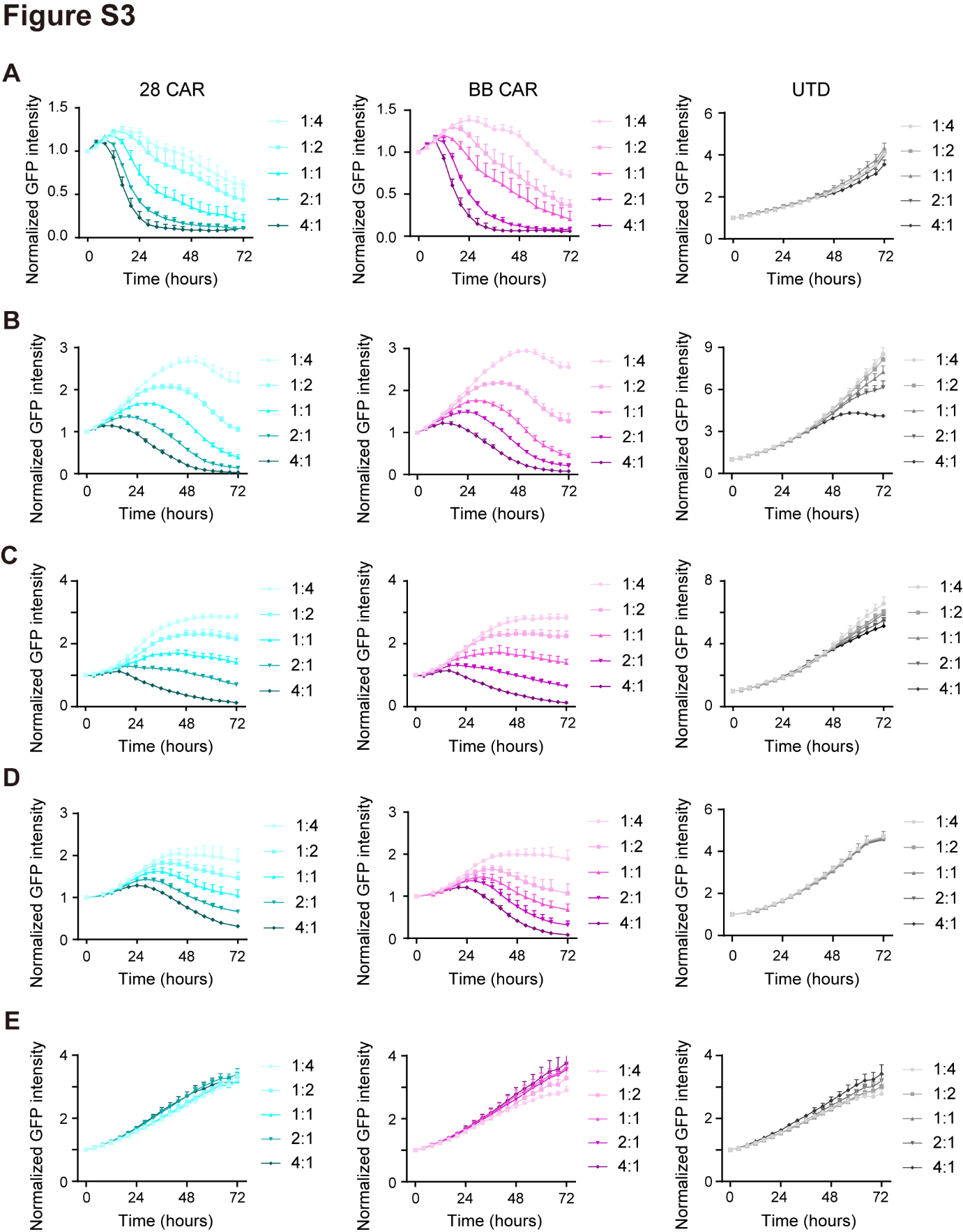


**Figure S3. Cytotoxic assays of EpCAM-specific CAR-T cells *in vitro*.**

Cytotoxic activity of UTD and two anti-EpCAM CAR-T cells (left: 28 CAR; middle: BB CAR; right: UTD) against GFP positive BxPC-3 (A), Capan-2 (B), CFPAC-1 (C), AsPC-1 (D) and hTERT-HPNE (E) cells coculture for 72 hours at E:T ratios of 4:1, 2:1, 1:1, 1:2 and 1:4. Data are presented as mean with SD of triplicate wells. *N* =1 T-cell donor.


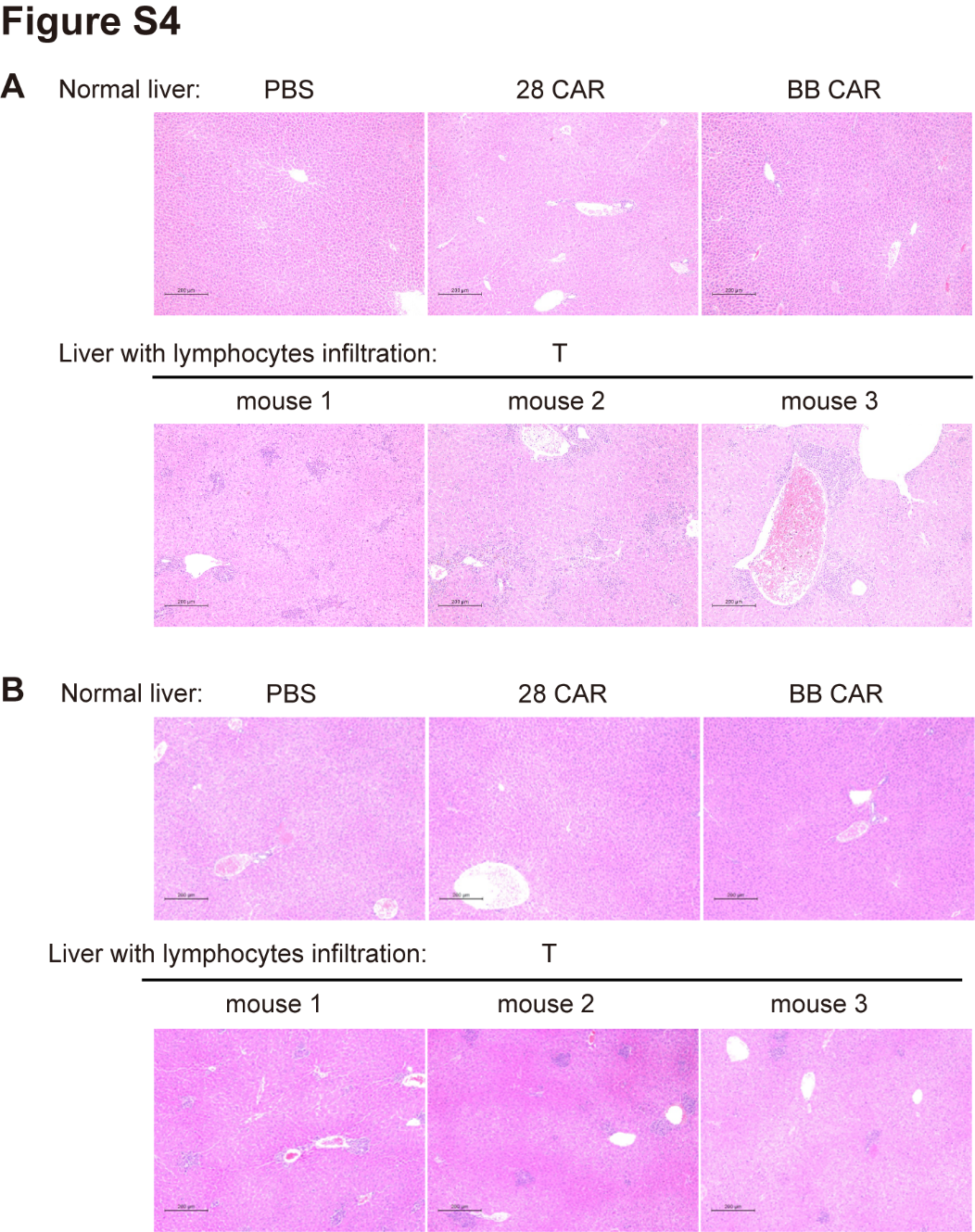


**Figure S4. Pathological signs of liver damage in the control T cell group but not CAR-T cell groups in xenograft mouse models of pancreatic cancer.**

Representative hematoxylin and eosin (H&E) staining images in xenograft mouse models of BxPC-3 (A) and Capan-2 (B) of normal liver in the PBS, 28 CAR and BB CAR-T cell group, as well as the liver with lymphocytes infiltration in the T cell group. Scale bars, 200 μm.

**Table S1. Cell lines, plasmids and antibodies used in this study**

| **REAGENT or RESOURCE** | **SOURCE** | **Additional information** |
| --- | --- | --- |
| **Cell Lines** | | |
| HEK293T | ATCC | For lentivirus packaging |
| J-N/N | Si, et al. (2023) | For CAR signaling activation |
| AsPC-1 | ATCC | EpCAM-positive target cell |
| BxPC-3 | ATCC | EpCAM-positive target cell |
| CFPAC-1 | ATCC | EpCAM-positive target cell |
| Capan-2 | Procell | EpCAM-positive target cell |
| HPAF-II | ATCC | EpCAM-positive target cell |
| SW1990 | Cell Resource Center, Peking Union Medical College (which is part of the National Science and Technology Infrastructure, the National Biomedical Cell-Line Resource, NSTI-BMCR. [http://cellresource.cn](http://cellresource.cn/)） | EpCAM-positive target cell |
| hTERT-HPNE | ATCC | EpCAM-negative target cell |
| AsPC-1-G-L | This study | EpCAM-positive target cell |
| BxPC-3-G-L | This study | EpCAM-positive target cell |
| CFPAC-1-G-L | This study | EpCAM-positive target cell |
| Capan-2-G-L | This study | EpCAM-positive target cell |
| hTERT-HPNE-GFP | This study | EpCAM-negative target cell |
| hTERT-HPNE-RFP | This study | EpCAM-negative target cell |
| Human PBMC | MILESTONE | Cat#PB050C |
| **Plasmids** | | |
| psPAX2 | MiaoLing | For viral packaging |
| pMD2.G | MiaoLing | For viral packaging |
| pHQR1.1-SP-EpCAM scFv-CD8α hinge and TM-CD28-CD3z | This study | Lentiviral vector of 28 CAR |
| pHQR1.1-SP-EpCAM scFv-CD8α hinge and TM-41BB-CD3z | This study | Lentiviral vector of BB CAR |
| pCDH-CMV-Luciferase-EF1α-copGFP-T2A-PuroR | This study | Lentiviral vector of firefly luciferase and GFP, fluorescent reporter of EpCAM-positive target cells |
| pHQR1.0-copGFP | This study | Lentiviral vector of GFP, fluorescent reporter of hTERT-HPNE |
| pHQR1.0-mRFP1 | This study | Lentiviral vector of RFP, fluorescent reporter of hTERT-HPNE in mixed spheroid model |
| **Antibodies** | | |
| CD326 (EpCAM) Monoclonal Antibody (1B7), PE | eBioscience | Cat#12-9326-42;  Clone: 1B7 |
| Anti-G4S linker (B02H1) mAb (PE) | PreScience | Cat#GS-ARPE100;  Clone: B02H1 |
| APC Anti-human CD69 | BioLegend | Cat#310910;  Clone: FN50 |
| CD3 Monoclonal Antibody (OKT3), Super Bright 600 | eBioscience | Cat#63-0037-41;  Clone: OKT3 |
| CD4 Antibody, anti-human, VioGreen | Miltenyi Biotec | Cat#130-113-230;  Clone: REA623 |
| CD8 Antibody, anti-human, APC-Vio770 | Miltenyi Biotec | Cat#130-110-681;  Clone: REA734 |
| CD62L Antibody, anti-human, PE-Vio770 | Miltenyi Biotec | Cat#130-113-621;  Clone: 145/15 |
| CD45RO Antibody, anti-human, APC | Miltenyi Biotec | Cat#130-113-556;  Clone: REA611 |

**Supplementary videos：**

Supplementary video 1. The killing dynamics of 28 CAR-T cells at the E:T ratio of 4:1 in the single tumoroid model.

Supplementary video 2. The killing dynamics of BB CAR-T cells at the E:T ratio of 4:1 in the single tumoroid model.

Supplementary video 3. The killing dynamics of control T cells at the E:T ratio of 4:1 in the single tumoroid model.

Supplementary video 4. The killing dynamics of 28 CAR-T cells at the E:T ratio of 2:1 in the single tumoroid model.

Supplementary video 5. The killing dynamics of BB CAR-T cells at the E:T ratio of 2:1 in the single tumoroid model.

Supplementary video 6. The killing dynamics of control T cells at the E:T ratio of 2:1 in the single tumoroid model.

Supplementary video 7. The killing dynamics of 28 CAR-T cells at the E:T ratio of 4:1 in the mixed tumoroid model (merged images of GFP and RFP).

Supplementary video 8. The killing dynamics of BB CAR-T cells at the E:T ratio of 4:1 in the mixed tumoroid model (merged images of GFP and RFP).

Supplementary video 9. The killing dynamics of control T cells at the E:T ratio of 4:1 in the mixed tumoroid model (merged images of GFP and RFP).

Supplementary video 10. The killing dynamics of 28 CAR-T cells at the E:T ratio of 4:1 in the mixed tumoroid model (GFP only).

Supplementary video 11. The killing dynamics of BB CAR-T cells at the E:T ratio of 4:1 in the mixed tumoroid model (GFP only).

Supplementary video 12. The killing dynamics of control T cells at the E:T ratio of 4:1 in the mixed tumoroid model (GFP only).
